# Death related to *Cedecea lapagei* in a soft tissue bullae infection: a case report

**DOI:** 10.1186/s13256-018-1866-x

**Published:** 2018-11-03

**Authors:** Victor Ramzes Chavez Herrera, Maria Fernanda Rosas De Silva, Homero Orendain Alcaraz, Gabriel Ceja Espiritu, Karla Carrazco Peña, Valery Melnikov

**Affiliations:** 1 Instituto Mexicano de Seguro Social, General Hospital Zona #1, University of Colima, Faculty of Medicine, Colima, Mexico; 2 Instituto Mexicano de Seguro Social, General Hospital Zona #1 , Colima, Mexico; 3 Faculty of Medicine, University of Colima, Colima, Mexico

**Keywords:** Hemorrhagic bullae, *Cedecea lapagei*, *Cedecea*, Soft tissue infection

## Abstract

**Background:**

*Cedecea lapagei* bacterium was discovered in 1977 but was not known to be pathogenic to humans until 2006. In the medical literature there are very few clinical case reports of *Cedecea lapagei*; none have reported a catastrophic death secondary to a soft tissue hemorrhagic bullae infection. As well as soft tissue infection, rare cases of pneumonia, urinary tract infections, peritonitis, osteomyelitis, bacteremia, and sepsis have been documented with the majority having good outcomes. Here, we present the first case of a fatal outcome in a *Cedecea lapagei* soft tissue infection with multiple hemorrhagic bullae.

**Case presentation:**

A 52-year-old Mexican man with antecedents of liver cirrhosis and treated hypertension was brought to our institution with clinical signs of sepsis and 16 to 18 hours of history of pain and edema in his right lower limb. During the course of the first day hospitalized in our institution, he developed several large serohematogenous bullae with ascending progression on his entire right lower limb. He subsequently developed multiple organ failure and septic shock with rapid deterioration, dying on the second day. Bullae fluid samples taken the first day undoubtedly isolated *Cedecea lapagei* within the second day using MicroScan WalkAway® 96 plus System as well Gram-negative bacteria in MacConkey and blood agar.

**Conclusions:**

The isolation of *Cedecea lapagei* was an unexpected etiological finding that will enable physicians in the future to consider this bacterium as a probable cause of serohematogenous bullae infections. We do not exclude contamination although it has never been isolated in bullae fluid in the medical literature. Future encounters with this bacterium should not be taken lightly as it may have the potential to have fatal outcomes.

## Background

The genus *Cedecea* (named after the abbreviation of its discovery site, the “CDC”, Centers for Disease Control) belongs to the family *Enterobacteriaceae*. *Cedecea* consists of six species. Three of these species are known human pathogens: *Cedecea davisae* (named after the bacteriologist, Betty Davis)*, Cedecea lapagei* (named after the British bacteriologist, Stephen Lapage), and *Cedecea neteri* (named after the microbiologist, Erwin Neter) [1]. Such names were proposed by Grimont *et al*. in 1980 [2]. Although discovered in 1977, it was not until the year 2006 that the species *Cedecea lapagei* became known as a pathogenic bacterium [1].

Because the discovery of *Cedecea lapagei* is relatively recent, and rarely diagnosed, there are few reported cases [1, 3–9]. As well as soft tissue infection, scarce known cases of pneumonia, urinary tract infections, peritonitis, osteomyelitis, bacteremia, and sepsis have been documented [1, 3, 6–9]. To the best of our knowledge, there do not appear to be any reported cases of a catastrophic hemorrhagic bullae soft tissue infection in which the primary underlying etiology was *Cedecea lapagei* bacteria that swiftly evolved into septic shock and abrupt death [3].

## Case presentation

We present the case of a 52-year-old Mexican man who worked as an office employee and lived in a suburban area of the city of Colima, Mexico. He and his wife denied recent trips outside the city. Zoonosis was absent. He was not physically active and did not have an adequate diet. Pathological antecedents revealed liver cirrhosis, diagnosed 12 years ago, alongside esophageal varices that had been treated with sclerotherapy 7 years prior. He also had a diagnosis of essential hypertension, diagnosed 20 years ago. His treatment prior to hospitalization included 20 mg of propranolol every 12 hours, which was used to treat his essential hypertension, esophageal varices, and to reduce his portal hypertension.

He arrived at our institution “IMSS General Hospital Zone 1, Colima” in the early afternoon (day 1, see Table 1). He began to experience extreme pain localized in his right foot, 16 to 18 hours prior to admission, with a local pain scale of 10/10. He denied any recent forms of punctures to the overlying skin (including animal and insect bites).

**Table 1:** Timeframe

To aid comprehension “Day 1”: begins during patient’s admission and ends 23.59 hours of that same day. “Day 2”: Begins during the end of day 1 and ends with the patient’s death.	

**Fig. 1:**
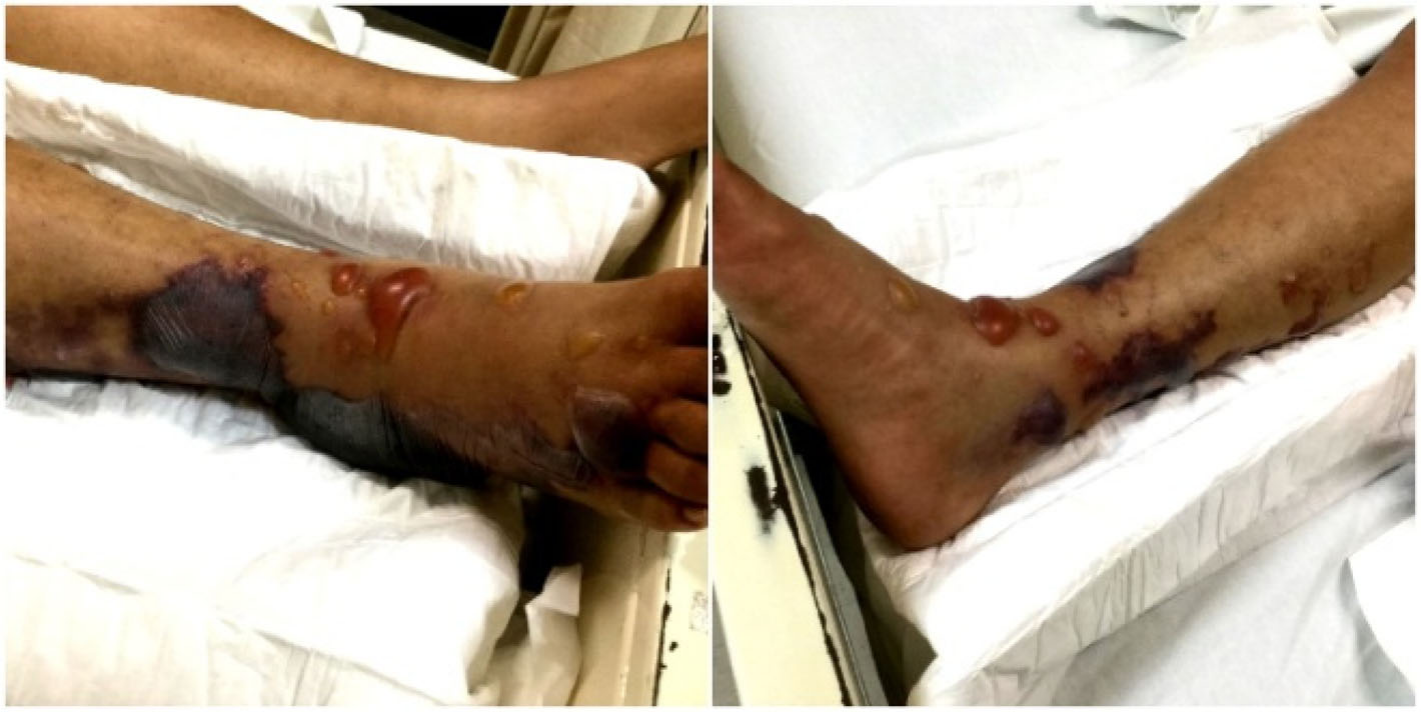
Hemorrhagic bullae on right lower limb

On physical examination during admission, he was somnolent, oriented in person and space, but not oriented to time. His vital signs were: arterial pressure (AP, systolic/diastolic) 67/49, mean AP (MAP) 55, heart rate (HR) 88, respiratory rate (RR) 16, and body temperature 36 °C. He presented hepatopathy facies and spontaneous eyelid opening, his oral mucosa was dry, and his neck showed jugular engorgement grade I. Both hemithorax were slightly hypoventilated with no adventitious sounds. Precordial was rhythmic with low intensity sounds and no murmurs were heard. Peristalsis was present in his abdomen but low in intensity and there was no hepatosplenomegaly. His upper extremities were symmetrical, eutrophic, with no signs of edema; he moved his upper extremities freely without any limitation, no asterixis was present, and a force scale of 4/5 was seen. During exploration of inferior extremities, there was a clear asymmetric pattern. His right lower extremity was volume augmented with signs of edema ++ including large and small bullae formation with serohematogenous liquid inside involving most of his right foot and ankle (Fig. 1). The pedal pulse was present but weak and had a local elevated temperature on palpation. His movements were markedly limited due to extreme pain. Deep vein thrombosis maneuvers were performed and were not present. Plantar reflexes were also not seen.

A peripheral intravenously administered high-dose double scheme of antibiotics (clindamycin plus ceftriaxone), crystalloid fluids, and corticosteroids was initiated. Laboratory studies were performed soon after admission: hemoglobin (Hb) 12.6 g/dl, hematocrit (Htc) 40.3%, mean corpuscular volume (MCV) 103.9 fL, white blood cells (WBC) 39,400/mm^3^, neutrophils 37,460/mm^3^, platelets 104,000/mm^3^, thrombin time (TT) 26.7 seconds, international normalized ratio (INR) 2.15, partial thromboplastin time (PTT) 42.8 seconds, glucose 61 g/dL, urea 102.72 mg/dL, creatinine 1.8 mg/dL, total bilirubin (TB) 3.4 mg/dL, direct bilirubin (DB) 2.6 mg/dL, indirect bilirubin 0.8 mg/dL, albumin 1.5 g/dL, alanine aminotransferase (ALT) 35 U/L, aspartate aminotransferase (AST) 58 U/L, P 7.1 mEq/L, Ca 8.4 mEq/L, Cl 106 mEq/L, K 6.4 mEq/L, Na 129 mEq/L, Mg 1.72 mEq/L, lactate dehydrogenase (LDH) 420 U/L, and C-reactive protein (CRP) 16.07 mg/L. Correction of hypoglycemia and electrolyte imbalance was initiated. Deep vein thrombosis was ruled out by clinical assessment and Doppler ultrasound. Aspiration of bulla liquid was obtained and sent to a laboratory for cultivation (positive to *Cedecea lapagei,* results returned on day 2). Interdisciplinary consultation with angiology was carried out (no indications were added). He was classified with a Sequential Organ Failure Assessment (SOFA) score of 11, Acute Physiology and Chronic Health Evaluation (APACHE) II of 22 points (42.4% mortality rate), and a Laboratory Risk Indicator for Necrotizing Fasciitis (LRINEC) score of 11 points (high risk > 75% of having necrotizing fasciitis) [10].

In the late afternoon (day 1) his somnolence and disorientation persisted, he was hypotensive with an AP of 80/40 with a MAP of 55, and he was unresponsive to fluid resuscitation. His hypoglycemia persisted despite vigorous treatment. No fever was present and his skin was pale. His left lower limb had ascending progression of serohematogenous bullae proximal to the patella. His extreme pain continued. Since our patient’s MAP was persistently below 65 mmHg despite intravenously administered fluid therapy, an anterior jugular central venous catheter was placed and we decided to include norepinephrine 8 mg in 0.9% 1000 cc physiological solution passing at a rate of 8 ml/hour, categorizing the case as septic shock [11]. Concomitantly he had developed acute kidney injury (AKI) “Kidney Disease: Improving Global Outcomes” (KDIGO) stage II.

During the night (day 1), abundant large fetid serohematogenous bullae and edema continued to advance up his right thigh with persistent generalized hypoperfusion. Because of previous mentioned conditions, it was decided to change antibacterial therapy to intravenously administered imipenem and clindamycin. Bullae fluid culture results and antibiogram were not available at that time. Norepinephrine was boosted to maintain MAP above 65 mmHg. Intensive care unit (ICU) was called in for valorization to admit our patient. Unfortunately, due to the lack of space in the ICU, this idea was dismissed. Since rapid deterioration was evident, new laboratory studies were ordered (approximately 10 hours after initial blood test): Hb 10.1 g/dL, Htc 32.7%, MCV 107.5 fL, WBC 11.6/mm^3^, platelets 35,000/mm^3^, TT “Does not coagulate”, PTT “Does not coagulate”, glucose 30 g/dL, urea 115.5 mg/dL, creatinine 2.4 mg/dL, K 6.4 mEq/L, Na 130 mEq/L, LDH 406 U/L, and CRP 220 mg/L. His SOFA score was 16.

Necrotizing fasciitis was our primary diagnosis due to clinical and laboratory findings (LRINEC score of 12 points). Even though the diagnosis was very likely, the lack of a biopsy study meant that we could not confirm such a diagnosis. Multiple organ dysfunction secondary to septic shock was also diagnosed. The differential diagnosis of animal or insect bite was dismissed due to lack of exposure history and lack of puncture wound.

During the next morning (day 2) he maintained the same general conditions. Norepinephrine was boosted up again (MAP 50). Soon after, he entered cardiopulmonary arrest and cardiopulmonary resuscitation was performed. Unfortunately he died later that morning.

## Discussion

*Cedecea* consists of Gram-negative, catalase-positive, oxidase-negative, lactose-negative, motile, nonsporing, nonencapsulated bacilli. The *Cedecea* bacteria are closely related to the *Serratia* bacteria but different in that *Cedecea* does not hydrolyze deoxyribonucleic acid (DNA) or gelatin [2].

*Cedecea lapagei* can be distinguished from other *Cedecea* strains by its ability to grow in media lacking thiamine [12]. It was not until 2006 that *Cedecea lapagei* was recognized as a potential pathogen to humans. It was first described in a case study involving a 55-year-old man in ambulatory peritoneal dialysis with hypertension and a recent liver transplant secondary to cirrhosis. He subsequently developed peritonitis and the culture from the peritoneal fluid isolated *Cedecea lapagei* indicating sensitivity to multiple antibiotics. He was treated with intraperitoneally and intravenously administered antibiotics which concluded with a complete recovery [1].

Although *Cedecea* itself is a rare bacterium, even fewer cases of *Cedecea lapagei* have been reported. None of the reported cases involved necrotizing fasciitis or a fatal soft tissue related infection. An extensive web research was performed using PubMed, MEDLINE, and Google databases,; the keywords used were “*Cedecea lapagei* death”, “*Cedecea lapagei* fatal”, “*Cedecea lapagei* necrotizing fasciitis,” and “*Cedecea lapagei* bulla” with all searches returning negative results.

Only one case report documented a fatal outcome involving *Cedecea lapagei.* Yetkin *et al.* reported a Turkish male diagnosed with subarachnoid hemorrhage [3]. While hospitalized he developed pneumonia caused by *Cedecea lapagei*; however, he did not die due to the infection as he responded positively to antibiotics but subsequently died as a result of his underlying base pathology [3].

The majority of case reports related to ours have narrated good outcomes and favorable responses to antibiotics [1, 6–9, 13, 14]. Antibiograms were performed in such cases, revealing an invariable resistance pattern. Resistance to ampicillin was met in most of the reported cases, including this one. Resistance to imipenem was not seen in most studies, but was in fact seen in our antibiogram. Not leaving it out may have contributed to our patient’s outcome [1, 6–9, 13] (Table 2).

**Table 2:** *Cedecea lapagei* antibiogram

Isolate: “*Cedecea lapagei*”	
Antibiotic	S/R/I (MIC)
Ampicillin/sulbactam	I (16/18)
Amikacin	S (≤ 4)
Ampicillin	R (> 16)
Aztreonam	S (≤ 8)
Ceftriaxone	S (≤ 8)
Ceftazidime	S (≤ 1)
Cefazolin	R (≥ 16)
Cefotaxime	S (≤ 2)
Ciprofloxacin	S (≤ 1)
Cefepime	S (≤ 2)
Cefuroxime	S (≤ 4)
Cefotetan	S (≤ 16)
Gentamicin	S (2)
Imipenem	R (≥ 8)
Levofloxacin	S (≤ 2)
Meropenem	S (≤ 4)
Moxifloxacin	S (≤ 2)
Piperacillin/tazobactam	S (≤ 8)

*I* intermediate, *MIC* minimal inhibitory concentration, *R* resistant, *S* sensitive

Another documented soft tissue infection case that involved *Cedecea lapagei* was reported by Salazar *et al.* [13]. Salazar *et al.* described a 28-year-old man who presented loss of tissue in his left foot due to an automobile accident. During hospitalization his limb evidenced a poor evolution, showing signs of infection. A culture from the lesion was drawn, demonstrating isolation of *Cedecea lapagei* which was susceptible to multiple antibiotics and resistant to ampicillin. The outcome in that case was favorable with antibiotic therapy [13]. In 2015, Biswal *et al.* described a case report involving a 50-year-old man with squamous cell carcinoma of the buccal mucosa [4]. He developed a pus-forming ulcer that isolated *Cedecea lapagei* in cultures. The bacterium was only resistant to ampicillin. He exhibited a vast improvement with antibiotic therapy [4]. Dalamaga *et al*. identified a *Cedecea lapagei* bacteremia infection from a wound infection in a patient suffering from cement-related chemical burns [5]. The patient’s left knee was erythematous with vesicles and ulcerations. Wound cultures were performed with positive results and fortunately ended with clinical success due to sensitive antibiotics [5]. *Cedecea lapagei* infections other than soft tissue infections have been documented [1, 3, 6–9].

Necrotizing fasciitis is a rare and deadly infectious disease. No cases of *Cedecea lapagei* (or others from the *Cedecea* genus) have been identified as causing this violent disease. Although a biopsy in our case was not obtained during hospitalization due to institutional limitations, the clinical and LRINEC score make the diagnosis very likely. However, similar bacteria such as *Serratia* have been detected that have disastrous fatal outcomes involving lower limbs [15], thus demonstrating that bacteria closely related to *Cedecea* could cause similar infections. Likewise, multiple pathogens have also been reported in lower limbs [16]. Not only is necrotizing fasciitis treacherous, but a Gram-negative monobacterial presentation has a higher susceptibility to septic shock and hemorrhagic bullae, much like in our case [17]. Despite this pathology, there are other infectious diseases which form large bullae that are linked with poor outcomes. In a case report from Yang *et al.*, a 69-year-old Chinese man developed septic shock due to bullae erysipelas-like lower limb infection, a needle aspirate of the bulla fluid was cultivated that showed a positive result for *Pseudomonas aeruginosa* [18].

Despite the fact that there has never been a documented isolation of this pathogen in bulla fluid, contamination is not excluded. Pande *et al*. demonstrated levels of these bacteria as an environmental reservoir. The *Cedecea* genus was found in 0.7% [19]; this means that our patient could have developed a community acquired environmental inoculation and adding the fact that he was immunocompromised. The bulla that was aspirated was not punctured before, which lowers the odds of contamination. We utilized the MicroScan WalkAway® 96 plus System that undoubtedly identified the microorganism *Cedecea lapagei* (99.99%); MacConkey and blood agar grew Gram-negative bacilli. Further diagnostic and therapy assessments could not be achieved due to the rapid evolution and lack of time, without forgetting our institution’s limitations.

## Conclusions

In light of our recent encounter with *Cedecea lapagei* this bacterium revealed itself in a whole new clinical manner. Early detection and correct treatment should always be kept in mind as it has demonstrated good outcomes. In our case, the isolation of this rare bacterium was an unexpected diagnostic feature that evidenced a devastating outcome. We encourage readers to not take this pathogen lightly as it may have the potential to be fatal. Not forgetting that this bacterium is relatively new, and many facts are still unknown that require further research.
